# Auditory central pathways in children and adolescents with multiple sclerosis

**DOI:** 10.1055/s-0043-1775985

**Published:** 2023-10-18

**Authors:** Dayane Aparecida Nascimento Barbosa, Liliane Aparecida Fagundes Silva, Alessandra Giannella Samelli, José Albino da Paz, Carla Gentile Matas

**Affiliations:** 1 Universidade de São Paulo, Faculdade de Medicina, Departamento de Fisioterapia, Fonoaudiologia e Terapia Ocupacional, São Paulo SP, Brazil.; 2 Universidade de São Paulo, Faculdade de Medicina, Instituto da Criança, Unidade de Neurologia, São Paulo SP, Brazil.

**Keywords:** Multiple Sclerosis, Hearing, Electrophysiology, Evoked Potentials, Auditory, Central Nervous System, Esclerose Múltipla, Audição, Eletrofisiologia, Potenciais Evocados Auditivos, Sistema Nervoso Central

## Abstract

**Background** Multiple sclerosis (MS) is an inflammatory demyelinating disease. Auditory evoked potential studies have demonstrated conduction and neural processing deficits in adults with MS, but little is known about the electrophysiological responses in children and adolescents.

**Objective** to evaluate the central auditory pathway with brainstem auditory evoked potentials (BAEP) and long-latency auditory evoked potentials (LLAEP) in children and adolescents with MS.

**Methods** The study comprised 17 individuals with MS, of both sexes, aged 9 to 18 years, and 17 healthy volunteers, matched for age and sex. All individuals had normal hearing and no middle ear impairments. They were assessed with click-BAEP and LLAEP through oddball paradigm and tone-burst stimuli.

**Results** Abnormal responses were observed in 60% of electrophysiologic assessments of individuals with MS. In BAEP, 58.82% of MS patients had abnormal responses, with longer wave V latency and therefore longer III-V and I-V interpeak latencies than healthy volunteers. In LLAEP, 52.94% of MS patients had abnormal responses. Although statistical differences were found only in P2-N2 amplitude, MS patients had longer latencies and smaller amplitudes than healthy volunteers in all components.

**Conclusion** Children and adolescents with MS had abnormal BAEP responses, with delayed neural conduction between the cochlear nucleus and the lateral lemniscus. Also, abnormal LLAEP results suggest a decrease in neural processing speed and auditory sensory discrimination response.

## Resumo

**Antecedentes** A esclerose múltipla (EM) é uma doença inflamatória desmielinizante. Estudos com potenciais evocados auditivos têm demonstrado déficits de condução e processamento neural em adultos com EM, mas pouco se sabe sobre as respostas electrofisiológicas em crianças e adolescentes.

**Objetivo** avaliar a via auditiva central por meio dos potenciais evocados auditivos de tronco encefálico (PEATE) e dos potenciais evocados auditivos de longa latência (PEALL) em crianças e adolescentes com EM.

**Métodos** Foram avaliados17 indivíduos com EM, de ambos os sexos, com idades entre 9 e 18 anos, e 17 voluntários saudáveis, pareados por sexo e idade. Todos os indivíduos tinham audição normal sem alterações de orelha média. Os indivíduos foram avaliados por meio do PEATE com estímulo clique e do PEALL com paradigma de oddball e estímulo tone-burst.

**Resultados** Foram observadas alteração em 60% das avaliações dos indivíduos com EM. No PEATE, 58,82% dos pacientes com EM apresentaram alteração, com aumento da latência da onda V, e interpicos III-V e I-V aumentados em comparação aos voluntários saudáveis. No PEALL, 52,94% dos pacientes com EM apresentaram alteração. Embora diferenças estatísticas foram observadas apenas na amplitude P2-N2, os pacientes com EM apresentaram latências prolongadas e amplitudes menores em comparação aos voluntários saudáveis para todos os componentes.

**Conclusão** Crianças e adolescentes com EM apresentaram alteração das respostas do PEATE, com atraso de condução neural entre o núcleo coclear e o lemnisco lateral. Além disso, os resultados alterados do PEALL sugeriram uma diminuição na velocidade de processamento neural e de discriminação sensorial da audição.

## INTRODUCTION

Multiple sclerosis (MS) is a chronic autoimmune disease of the central nervous system – where inflammation, demyelination, and axonal loss occur as early as the initial stages of the disease. It is one of the most frequent causes of neurological disability in young individuals. 1 2

MS affects mainly young adults, mostly women 20 to 40 years old. However, an estimated 30,000 children and adolescents worldwide are believed to be affected by it, totaling 2% to 5% of all cases. 3

MS manifests as an inflammatory disease among children and youth, causing more seizures and evidence of brain atrophy, axonal damage, and accumulated lesions identified in magnetic resonance imaging (MRI) than in disease onset at adulthood. 4

Considering these individuals' neuronal impairment, it has been recommended to use evoked potentials in batteries to diagnose MS, assess the progress of the disease, and monitor the benefits and limitations of various treatments. 4 5 6 7 8 These potentials can measure the physiology of neurological changes, helping identify the disease locus and lesion severity, 7 though undetectable with MRI. 8 Studies have pointed out that assessments with auditory evoked potentials can locate lesions throughout the auditory pathways at a rate almost similar to that of MRI 6 –which is greatly important, as it is a noninvasive and low-cost procedure.

Auditory evoked potentials assess the neuroelectric activity in the auditory pathway from the auditory nerve to the cerebral cortex, evoked with acoustic stimuli. The brainstem auditory evoked potentials (BAEP) are one of the most used resources in clinical practice; their main objectives are to identify changes from the auditory nerve to the brainstem and estimate the electrophysiological hearing threshold. 9 In their turn, the long-latency auditory evoked potentials (LLAEP) reflect the neuroelectric activity of the auditory pathway in the thalamus and auditory cortex – which are structures that involve functions of discrimination, integration, and attention, providing information on the functioning of the central auditory nervous system. 10 11

Various studies have assessed electrophysiological measures in adult MS patients. 5 6 8 12 13 14 15 16 17 18 19 20 21 22 23 24 25 26 27 28 29 30 Despite the vast literature on the topic, studies investigating impairments in the central auditory nervous system of children and youth are scarce. Hence, this study aimed to assess the central auditory pathway with BAEP and LLAEP in children and adolescents with MS.

## METHODS

The study group (SG) comprised 17 individuals who attended the Children's Institute of the Medical School Clinics Hospital at the University of Sao Paulo (HCFMUSP), diagnosed with MS (according to criteria of the International Pediatric Multiple Sclerosis Study Group for pediatric MS), of both sexes (nine females and eight males), aged 9 to 18 years (13.71 ± 3.01). The age of symptom onset ranged from 4 to 16 years (11.71 ± 3.51), and the diagnosis was confirmed by 6 to 17 years old (12.29 ± 3.45).

The control group (CG) comprised a convenience sample of 17 healthy volunteers, matched with SG for age and sex, without developmental impairments or neurological or psychiatric complaints, recruited from local schools.

None of the participants had obstructions in the external auditory meatus or conductive impairments – they had type-A tympanograms –, and all of them had normal hearing (hearing thresholds below 15 dB HL at 500 to 4000 Hz).

The research was approved by the institution's Research Ethics Committee under number 1.784.31. All parents/guardians and participants respectively signed informed consent and assent forms before the study.

After the complete audiological assessment, the auditory evoked potentials were obtained using the Smart EP equipment manufactured by Intelligent Hearing System and ER 3-A insert earphones. During the assessment, subjects remained seated in a reclining chair, in an acoustically and electrically treated room. The skin surface of the forehead, mastoids, and scalp was cleaned with abrasive paste, and Ag-AgCl electrodes were then positioned with electrolytic paste and micropore tape, following the international 10-20 system (International Electrode System). Electrode impedance was maintained below 3 kOhms in all trials.

Two BAEP channels were applied using an electrode montage of Fz (active electrode), Fpz (ground), and M1 and M2 (reference electrodes). This potential was evoked monaurally through rarefaction click, at a presentation rate of 19.1 clicks per second, at 80 dBnHL, using a 100 Hz high-pass filter, 1500 Hz low-pass filter, and 12 ms recording window. Dual trials were performed with 2,048 sweeps each to check reproducibility.

Waves I, III, and V were identified and analyzed regarding absolute latencies, and I-III, III-V, and I-V interpeak latencies. Based on the equipment's user manual, each person's BAEP results were classified as either normal or abnormal (when at least one of the ears was abnormal). Changes were classified as follows: changes in the low brainstem if there was an increase in the I-III interpeak latency; changes in the high brainstem if there was an increase in the III-V interpeak latency; or mixed if both I-III and I-V interpeak intervals had increased latencies.

For LLAEP, the electrode montage was Cz (active electrode), Fpz (ground), and M1 and M2 (reference electrode). Tone-burst stimuli were presented monaurally in an oddball paradigm, at 75 dBnHL, with the standard stimulus (85%) at 1000 Hz and the target stimulus (15%) at 2000 Hz. A total of 300 sweeps were presented at 1.1 sweeps per second, with high- and low-pass filters between 1 and 30 Hz, and a 500 ms recording window.

Participants were instructed to pay attention to the target stimuli and count aloud the number of times they occurred. The trial that corresponded to the target stimuli was subtracted from the standard stimuli. P1, N1, P2, and N2 components were identified and analyzed regarding latency and amplitude in the standard trial, whereas P3 was so in the target trial. P1, N1, P2, N2, and P3 latencies and P1-N1, P2-N2, and N2-P3 amplitudes were analyzed. The normality of absolute latencies followed that proposed by McPherson 11 for each age group.

Quantitative values were analyzed regarding descriptive analysis, and a no-paired t-test was used to compare SG and CG. Concerning qualitative data, the proportion of normal and abnormal results and the types of changes were analyzed with Fisher's exact test. Statistical significance was set at p-value ≤ 0.05 for all inferential analyses.

Also, an analysis was carried out in order to verify the association between the main focus of alteration on MRI (considering bridge, midbrain, cerebellar peduncles/cerebellum, and IV ventricle) and the results of BAEP and LLAEP, by means of Fisher's exact test.

## RESULTS

Absolute and interpeak BAEP latencies and absolute LLAEP latencies and amplitudes were initially compared between the right and left ears of each group. As none of the analyzed variables presented significant differences between the ears, the right and left ears were grouped for the other analyses (comparison between groups).

The comparison between SG and CG revealed statistically significant differences in BAEP III-V and I-V interpeak latencies, with longer latencies in SG ( Table 1 ). It is noteworthy that in BAEP, 70% of the 10 SG individuals with abnormal results had changes in the high brainstem, whereas 30% had them in the low brainstem.

**Table 1:** Absolute BAEP waves I, III, and V latencies and I-III, III-V, and I-V interpeak latencies of both groups

	Group	Mean (ms)	SD	p-value
Wave I	SG	1.54	0.13	0.172
	CG	1.59	0.07	
Wave III	SG	3.70	0.20	0.276
	CG	3.76	0.10	
Wave V	SG	5.69	0.22	0.123
	CG	5.59	0.14	
I-III interpeak interval	SG	2.17	0.14	0.822
	CG	2.18	0.11	
III-V interpeak interval	SG	1.97	0.22	0.015*
	CG	1.82	0.09	
I-V interpeak interval	SG	4.14	0.21	0.027*
	CG	4.00	0.13	

Abbreviations: BAEP, brainstem auditory evoked potentials; SG, study group; CG, control group; ms, milliseconds; SD, standard deviation. Note: *p-value with a statistically significant difference.

For LLAEP, the comparison between groups revealed statistically significant differences in P2-N2 amplitude, which was higher in CG ( Table 2 ). Furthermore, P1, P2, and N2 were the most abnormal components, with 66.7% of the changes.

**Table 2:** Absolute LLAEP waves P1, N1, P2, N2, and P3 latencies and P1-N1, P2-N2, and N2-P3 amplitudes of both groups

	Group	Mean	SD	p-value
P1 latency (in ms)	SG	65.50	23.62	0.186
	CG	56.59	13.52	
N1 latency (in ms)	SG	109.41	25.40	0.189
	CG	99.53	16.63	
P2 latency (in ms)	SG	177.91	32.02	0.179
	CG	165.79	17.42	
N2 latency (in ms)	SG	226.18	32.05	0.978
	CG	225.94	17.40	
P3 latency (in ms)	SG	308.65	26.81	0.303
	CG	318.97	30.63	
P1-N1 amplitude (in µV)	SG	4.76	2.87	0.598
	CG	5.27	2.72	
P2-N2 amplitude (in µV)	SG	3.49	2.28	0.025*
	CG	5.99	3.75	
N2-P3 amplitude (in µV)	SG	10.12	5.84	0.717
	CG	10.82	5.34	

Abbreviations: LLAEP, long-latency auditory evoked potentials; SG, study group; CG, control group; ms-milliseconds; µV, microVolts; SD, standard deviation. Note: *p-value with a statistically significant difference.

The comparison of normal and abnormal BAEP and LLAEP results between the groups showed a higher incidence of changes in SG than in CG, with a statistically significant difference between them ( Table 3 ).

**Table 3:** Distribution of normal and abnormal BAEP and LLAEP results of both groups

		Study group		Control group		p-value
		Sample number	Percentage (%)	Sample number	Percentage (%)	
BAEP	Abnormal	10	58.8%	0	0.0%	<0.001
	Normal	7	41.2%	17	100.0%	
LLAEP	Abnormal	9	52.9%	0	0.0%	<0.001
	Normal	8	47.1%	17	100.0%	
BAEP + LLAEP	Abnormal	6	60.0%	0	0.0%	0.001
	Normal	4	40.0%	17	100.0%	

Abbreviations: BAEP, brainstem auditory evoked potentials; LLAEP, long-latency auditory evoked potentials.

Moreover, the combination of BAEP and LLAEP in group comparison indicated that six individuals had changes in both potentials, four individuals had changed only in BAEP, and three individuals had changed only in LLAEP ( Table 3 ). Only four individuals presented normal results in both BAEP and LLAEP.

No association was observed between MRI results and BAEP and LLAEP results (p-value > 0.05).

## DISCUSSION

This study aimed to assess the central auditory pathways in children and adolescents with MS. This age range is seldom addressed in the literature, probably because the disease is more prevalent in adults.

BAEP analysis revealed changes in 58.82% of patients. Increased wave V and consequently in III-V and I-V interpeak intervals indicate decreased neural conduction speed of the acoustic stimuli in the auditory pathways in the high brainstem, between the cochlear nucleus and lateral lemniscus.

The scientific literature reports a great variability in the incidence of BAEP changes in adults with MS, encompassing 20%, 21 21.9%, 22 30%, 17 45%, 16 and 65% 15 of the cases. Such changes included morphology changes, abnormal tracing, increased absolute and interpeak latencies, and the absence of some waves. 17 27 Furthermore, Di Stadio et al. 31 conducted a literature review and concluded that 100% of MS patients had some type of BAEP change.

Studies in adults reported similar results to those found in the present one, with increased wave V latency, 19 25 32 III-V interpeak latency, 21 25 and I-V interpeak latency. 8 16 19 21 25 In addition, some studies also found increased latencies in waves I 8 and III 8 19 32 and in interpeak interval I-III. 8 19 21 25

As for children and youth, a study assessed a small group of 11 children and adolescents aged 9 to 17 years and found increased III-V and I-V interpeak latencies, suggesting changes in the high brainstem. 33 However, another study assessed 10 adolescents aged 13 to 17 years and reported increased latencies in waves III and V and increased interpeak intervals I-III and I-V, suggesting changes in the low brainstem. 34

Such results may suggest a gradual impairment of the auditory pathways, progressing from the most central region of the auditory system to future impairments in more distal regions of the central nervous system. Nevertheless, the results found in the literature remain quite variable. Moreover, there is a gap in the characterization of samples regarding MS locus and the time elapsed from the disease onset to the study. Hence, future studies that control these variables may find more systematic and consistent results concerning impairments in this population's auditory pathways.

As for the cortical auditory pathways, more than half (52.94%) of MS patients in this study had LLAEP changes. Even though there were no statistically significant differences in latency values, SG had longer latencies than the healthy volunteers. Similarly, there was a decrease in response amplitudes, although a statistically significant difference was found only in P2-N2.

Barbosa et al. 33 also found a significant decrease only in P2-N2 amplitude in children and adolescents with MS. On the other hand, regarding adults, there are reports of increased latencies in N1, 14 28 P2, 12 14 N2, 12 14 and P3, 5 12 14 18 26 28 as well as increased amplitudes in P2, 14 28 N2, 28 and P3. 14

These results suggest that MS patients may have slowed neural processing and decreased neural activity in sensory, inattentional, 5 and attentional discrimination of acoustic stimuli, due to demyelination 18 –which slows down conduction, while axonal degeneration attenuates the amplitude of the potential. 7

According to Comi et al., 35 demyelination may cause neural conduction attenuation, high-frequency impulse transmission failures, blocked conduction, and secondary axonal degeneration. Thus, abnormalities found in MS patients' evoked potentials may consist of delayed latencies in one or more components, morphological abnormalities, and an increased refractory period. None of these anomalies is specific to MS, but changes perceived in long-term follow-up may indicate the progress of demyelination.

In the present study, no association was observed between the main focus of alteration detected on MRI and the electrophysiological results. This result may be justified by the limited sample size, considering that the population is heterogeneous in terms of the different demyelinating lesion sites found in each patient.

In a larger sample, of 32 patients, abnormal latencies in the potentials have been related to the locus of demyelinating lesions, agreeing with what was observed in the MRI. 13 The combined assessment of short-, middle-, and long-latency auditory evoked potentials have shown an 87% sensitivity, helping detect and confirm MS locus. 15 Hence, evoked potential assessment has proved to be a resource available when MRI is not. It can be used to monitor treatment and long-term prognosis and to assess changes that are not yet evident or specific in MRI. 6 7 8 25 Furthermore, LLAEP has been correlated with disease duration 12 and neuropsychological test results. 14 24 These data furnish information on the application of LLAEP to assess the degree of cognitive impairment and investigate the neural origin of the disease. 5

Changes in temporal resolution and auditory task memory and difficulties discriminating speech in noisy environments have been described in MS 23 –which may justify the decreased P2-N2 amplitude. Moreover, some cognitive function impairments may be related to attention, processing speed, working memory, visuospatial skills, and executive functions. 20 36 37 Such deficits can interfere with academic and social performance and the self-perceived capacity to do everyday tasks, therefore, detecting it immediately is essential to the treatment.

MS impact on cognitive functions is still little known – although changes in cognitive functions are known to be common in children with MS. 38 39 Since this population attends school – a phase when auditory processing complaints are frequent even in individuals with no other impairments –, special attention must be paid to ensure adequate treatment and resources to make hearing easier in the classroom or other settings where listening is difficult, thus favoring learning and better quality of life.

Various otorhinolaryngological symptoms are also described in MS, including speech disorders, sleep disorders, vertigo, imbalance, dysphagia, changes in smell, and hearing loss. 40 These data, along with the present study's findings, make clear the importance of otorhinolaryngological and speech-language-hearing follow-ups on children and adolescents with MS.

This study had a larger sample than the previous one that assessed auditory evoked potentials in same-age MS patients. Nonetheless, the present research had a limited sample size, which hindered other correlations concerning, for instance, the influence of age on symptom onset, disease duration, and medications used. Therefore, future research is expected to have larger samples and characterize them in further detail to control other variables that might influence electrophysiological responses.

Another limitation of the study, regarding LLAEP analysis, was that it did not obtain data on the participants' school achievements. Neither was it possible to perform a behavioral assessment of the central auditory processing or a neuropsychological assessment battery to correlate with the findings of the electrophysiological assessment. Thus, future studies with larger samples that complement such data may clarify other nuances that could not be measured in the present one.
